# Diffusion-Weighted Magnetic Resonance Imaging and Apparent Diffusion Coefficient Mapping Detect Kidney Infiltration in Acute Myeloid Leukemia: A Case Report

**DOI:** 10.1016/j.xkme.2025.101226

**Published:** 2025-12-16

**Authors:** Yusuke Yoshimura, Yuki Oba, Kei Kono, Hisashi Yamamoto, Hisashi Kamido, Hisashi Sugimoto, Shigekazu Kurihara, Masayuki Yamanouchi, Tatsuya Suwabe, Kenichi Ohashi, Yoshifumi Ubara, Naoki Sawa

**Affiliations:** 1 Nephrology Center, Toranomon Hospital Kajigaya, Kanagawa, Japan; 2 Department of Pathology, Toranomon Hospital, Tokyo, Japan; 3 Department of Hematology, Toranomon Hospital, Tokyo, Japan

**Keywords:** acute myeloid leukemia, kidney infiltration, diffusion-weighted magnetic resonance imaging, apparent diffusion coefficient, kidney biopsy

## Abstract

We describe a case of 70-year-old man with type 2 diabetes and myelodysplastic syndrome progressing to acute myeloid leukemia (AML) who presented with rapidly worsening kidney function. Although computed tomography imaging of the patient’s kidneys was unremarkable, subsequent magnetic resonance imaging (MRI) showed high signal intensity on diffusion-weighted imaging and low signal intensity on the apparent diffusion coefficient map in the kidneys. A kidney biopsy from the region that exhibited abnormal findings on MRI showed blast cell infiltration within the kidney capsule and proximal tubules. After initiating venetoclax and azacitidine therapy, the patient’s AML promptly improved, and his kidney function rapidly recovered. Kidney infiltration in AML is rare and difficult to diagnose because of nonspecific symptoms and the limited sensitivity of plain computed tomography. This case highlights the value of MRI, especially diffusion-weighted imaging and apparent diffusion coefficient mapping, for the detection of blast cell infiltration in the kidneys. To our knowledge, this is the first case in which kidney infiltration was identified in a patient with AML based on imaging findings. This case supports the consideration of MRI as a noninvasive diagnostic tool in patients with unexplained kidney dysfunction and hematologic malignancies.

Approximately 0.5% of patients with acute myelogenous leukemia (AML) present with kidney lesions accompanied by kidney dysfunction.^1^ However, case reports suggest that many patients are asymptomatic, and plain computed tomography (CT) may have limited sensitivity for detecting infiltrative disease of the kidney, making the prompt diagnosis difficult.^2^, ^3^, ^4^, ^5^, ^6^ However, some cases lead to severe conditions such as acute kidney injury (AKI) requiring dialysis.^3^^,^^4^^,^^6^ The kidney dysfunction in patients with AML is associated with significant morbidity and mortality, and early treatment can lead to improved patient outcomes.^3^^,^^7^ Thus, more sensitive diagnosis techniques are needed.

Magnetic resonance imaging (MRI), specifically diffusion-weighted imaging (DWI) and apparent diffusion coefficient (ADC) mapping, can identify areas of increased cellularity.^8^ Recently, we reported that DWI is highly sensitive for detecting immunoglobulin G4-related tubulointerstitial nephritis (IgG4-TIN) lesions and that MRI findings can predict the degree of fibrosis in IgG4-TIN.^9^^,^^10^

Based on these findings, we hypothesized that DWI and ADC mapping might also help identify the kidney infiltration in patients with AML, given its high cellularity. We confirmed kidney infiltration in a patient with AML by taking a biopsy from a lesion identified on DWI that showed massive blast infiltration.

## Case Report

A 70-year-old man diagnosed with AML presented with a history of rapidly worsening kidney function. His prior creatinine (Cr) level was approximately 1.0 mg/dL, and his qualitative urine protein tests had consistently been negative at least until a health checkup conducted 6 months before the diagnosis of AML. However, over the last 6 months, his Cr levels had rapidly increased to 2.04 mg/dL, and his spot urine protein-Cr ratio (UPCR) had increased to 0.89 g/gCr.

The patient had a 10-year history of type 2 diabetes mellitus, and his medications included 0.9 mg of voglibose, 5 mg of linagliptin, 4 mg of candesartan, and 10 mg of febuxostat. His blood glucose levels were generally well controlled. He was diagnosed with myelodysplastic syndrome at the age of 67 years, which subsequently progressed to AML at the age of 69 years. However, the patient refused standard chemotherapy and did not present with overt symptoms related to the malignancy.

The results of a blood examination on admission are shown in Table 1.

**Table 1:** Laboratory Data on Admission

	Data	Reference Range
Leukocyte, μL	14,400	3,300-8.600
Promyelocyte, %	0.5	-
Myelocyte, %	1.0	-
Metamyelocyte, %	0	-
Neutrophil, %	39.5	-
Eosinophil, %	1.5	0.0-8.5
Basophil, %	0	0.0-2.5
Monocyte, %	1.0	2.0-10.0
Lymphocyte, %	5.5	16.5-49.5
Atypical lymphocyte, %	0	-
Blast cell, %	51.0	-
Hemoglobin, g/dL	7.7	13.7-16.8
Platelet count, ×10^4^/mL	58.6	158-348
Total protein, g/dL	8.6	6.6-8.1
Albumin, g/dL	4.1	4.1-5.1
Aspartate aminotransferase, U/L	32	13-30
Alanine aminotransferase, U/L	21	10-42
LDH, U/L	546	124-222
Uric acid, mg/dL	6.6	3.7-7.0
UN, mg/dL	18	8-20
Cr, mg/dL	2.04	0.65-1.07
eGFR, mL/min/1.73 m^2^	26.3	-
C-reactive protein, mg/dL	0.63	<0.3
Hemoglobin A1c, %	5.5	4.9-6.0
Sodium, mEq/L	139	138-145
Potassium, mEq/L	4.3	3.6-4.8
Chloride, mEq/L	105	101-108
M protein	Negative	Negative
Free light chain		
Κ	108	3.3-19.4
Λ	107	5.7-26.3
κ/λ	1.01	0.26-1.65
Antinuclear antibody	<40	<40
Immunoglobulin G, mg/dL	3,128	861-1,747
Immunoglobulin A, mg/dL	462	93-393
Immunoglobulin M, mg/dL	126	33-183
Complement 3, mg/dL	85	73-138
Complement 4, mg/dL	16	17-45
Complement hemolytic 50, U/mL	56	31-58
Proteinase 3 antineutrophil cytoplasmic antibodies, IU/mL	<2.0	<2.0
Myeloperoxidase-specific antineutrophil cytoplasmic antibody, IU/mL	<3.5	<3.5
Hepatitis B surface antigen	Negative	Negative
Hepatitis B core antibody	Negative	Negative
Hepatitis C virus antibody	Negative	Negative
Red blood cell, /HPF	<1	<4
UPCR, g/gCr	0.89	-
α1-microglobulin, mg/L	55.3	1.0-17.8
β2-microglobulin, mg/L	25,219	14-329
N-acetyl-β-D-glucosaminidase, U/L	29	-

Abbreviations: Cr, creatinine; eGFR, estimated glomerular filtration rate; HPF, high power field; LDH, lactate dehydrogenase; UN, urea nitrogen; UPCR, urine protein-creatinine ratio.

**Figure 1:**
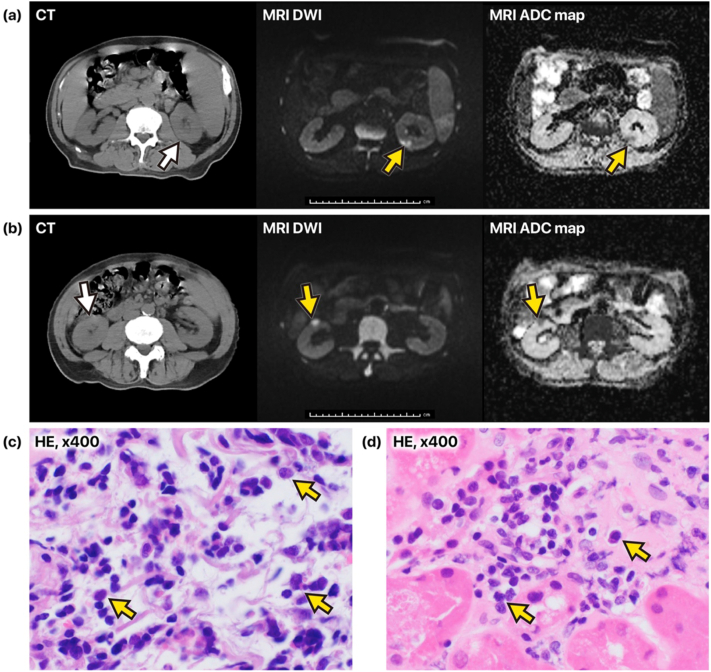
CT and MR images of AML with infiltration in the kidneys and kidney biopsy findings (A, B) The 3 images in the top row and the 3 images in the bottom row each show roughly the same cross-sectional planes. The images from left to right are from computed tomography (CT), magnetic resonance diffusion-weighted imaging (DWI), and apparent diffusion coefficient (ADC) mapping. The arrows indicate the location of the lesion. In all cross-sections, no obvious abnormalities were observed on CT; however, on MRI, mass-like lesions displaying high signal intensity on DWI and low signal intensity on the ADC map were clearly localized in the superficial kidney cortex. (C) Numerous atypical cells with irregularly contoured, enlarged nuclei extensively infiltrating the collagen fiber bundles within the kidney capsule (hematoxylin‒eosin stain; original magnification, ×400). (D) Atypical cells in peritubular capillaries (hematoxylin‒eosin stain; original magnification, ×400). Abbreviations: ADC, apparent diffusion coefficient; CT, computed tomography; DWI, diffusion-weighted imaging; HE, hematoxylin‒eosin, MRI, magnetic resonance imaging.

Fig 1A and B illustrates the discrepancy between the CT and MRI findings in the kidneys of this patient. Initial kidney imaging with CT was unremarkable. Owing to concerns regarding an infiltrative process, MRI was performed, which showed multiple mass-like lesions predominantly in the cortex of each kidney, presenting with high signal intensity on DWI and low signal intensity on the ADC map. These imaging findings suggested leukemic infiltration.

Based on the MRI findings, we strongly suspected that the lower pole of the left kidney contained a large lesion located in a position suitable for safe puncture. Therefore, ultrasound-guided kidney biopsy was performed on the mass lesion in the lower pole of the left kidney. Numerous atypical cells with irregularly contoured, enlarged nuclei had extensively infiltrated the collagen fiber bundles within the kidney capsule (Fig 1C), as well as the cortical interstitium, tubular epithelium, and peritubular capillaries (Fig 1D). Immunohistochemical analysis of these atypical cells in the kidney capsule showed positivity for myeloperoxidase and CD34, focal positivity for KIT, and negativity for terminal deoxynucleotidyl transferase, CD3, and CD20. These findings were consistent with blast cells.

A bone marrow aspirate and biopsy confirmed FMS-like tyrosine kinase 3 internal tandem duplication-positive AML. Based on these findings, atypical cells observed in the kidneys were confirmed as infiltrated blast cells of AML.

**Figure 2:**
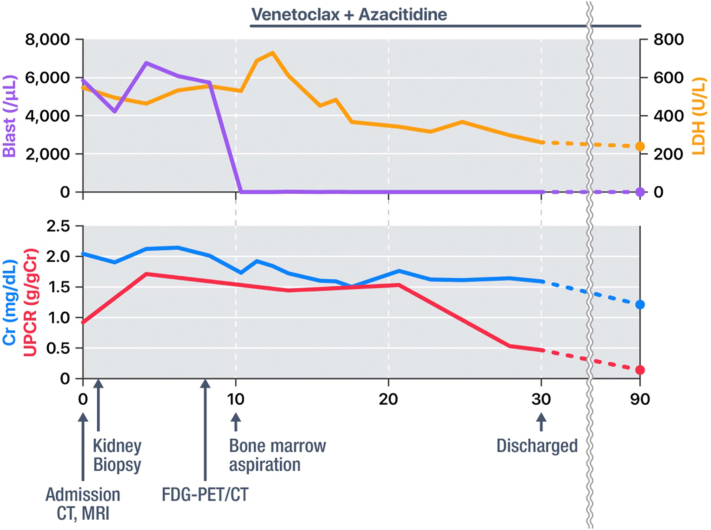
Clinical course. Abbreviations: Cr, creatinine; CT, computed tomography; FDG-PET/CT, fluorine-18 fluorodeoxyglucose positron emission tomography/computed tomography; LDH, lactate dehydrogenase; MRI, magnetic resonance imaging; UPCR, urine protein-creatinine ratio.

Fig 2 summarizes the time course of AML and kidney dysfunction. The patient received venetoclax and azacitidine therapy, which led to a rapid decrease in peripheral blood blast count. Following the improvement of AML, the patient’s serum Cr level decreased to 1.2 mg/dL, with a concomitant reduction in UPCR to 0.1 g/gCr.

## Discussion

Approximately 30%-50% of patients with leukemia or malignant lymphoma have kidney infiltrative lesions, and approximately 0.5% of cases progress to kidney dysfunction. However, the diagnosis of leukemic infiltration in kidneys is difficult.^1^^,^^11^ Although pathological examination remains the gold standard for confirming AML kidney infiltration, kidney biopsy is an invasive procedure. Therefore, imaging modalities capable of noninvasively detecting kidney infiltration in AML and guiding the decision for kidney biopsy are highly desirable.

Table 2 summarizes representative cases of kidney infiltration of AML. Among these, fluorine-18 fluorodeoxyglucose positron emission tomography/computed tomography (FDG-PET/CT) identified abnormal uptake in one case, whereas the other reports relied on ultrasound or plain CT, which often failed to detect lesions. These case studies suggests that plain CT, which is generally conducted for patients with AML, may have limited sensitivity for detecting infiltrative disease of the kidney. Contrast-enhanced CT raises concerns about the deterioration of kidney function in patients with existing kidney lesions. FDG-PET/CT has shown promise as a noninvasive method for detecting extramedullary disease in patients with AML. However, FDG-PET/CT is expensive, and there are only a limited number of facilities where it can be performed.^4^ The presence of kidney dysfunction in patients with AML is associated with significant morbidity and mortality, and the early initiation of antileukemia therapy can lead not only to improved kidney function but also to the remission of AML in patients with kidney infiltration.^3^^,^^7^ Thus, more sensitive techniques are needed to establish a diagnosis.

**Table 2:** Published Cases of Kidney Infiltration of AML

Author (y)	Age (y)/Sex	Kidney Function Changes	Imaging Modality and Kidney Findings	Kidney Biopsy Findings	Treatment	Kidney Outcome
Ooi et al^2^ (2013)	75/M	Worsened over 2 mo (Cr 350 μmol/L (∼3.96 mg/dL), proteinuria 1.06 g/d, no hematuria)	AUS: No abnormality	MPO positive leukemic infiltration in interstitium and vessels	Palliative care	Deceased within weeks
Aratani et al^3^ (2020)	43/F	AKI (Cr 0.59 → 3.08 mg/dL, proteinuria negative → 3+, hematuria negative → 1+)	Plain CT: Bilateral nephromegaly without detectable lesions	Leukemic infiltration confirmed	Idarubicin + cytarabine	Kidney function improved
	66/F	AKI (Cr 0.74 → 2.96 mg/dL, proteinuria negative → 2+, hematuria negative → 2+)	Plain CT: Bilateral nephromegaly without detectable lesions	No biopsy performed	Idarubicin + cytarabine	Kidney function improved
Chauhan et al^4^ (2021)	50/M	AKI (Cr 0.8 → 3.8 mg/dL, anuria)	FDG-PET/CT: Abnormal uptake observed bilaterally AUS and plain CT: Bilateral nephromegaly without detectable lesions	No biopsy performed	Hypomethylating agent + venetoclax	Morphologic and metabolic remission achieved, and Cr improved to 1.2 mg/dL
Aggarwal et al^5^ (2024)	60/M	AKI (Cr 5.96 mg/dL without urine abnormality)	AUS: Bilateral nephromegaly without detectable lesions	PAS and MPO positive leukemic infiltration in insterstitium and peritubular capillaries	NA	Deceased within 48 h because of leukostasis
Lee et al^6^ (2024)	67/M	AKI (Cr 1.02 → 5.47 mg/dL, proteinuria negative → 2.21 g/gCr)	Plain CT: No abnormality	Bowman’s capsule rupture with atypical cells infiltrating Bowman’s space Myeloblast-like cell infiltration in interstitium with tubular atrophy	Cytarabine + venetoclax	Cr improved to 1.5 mg/dL

Abbreviations: AKI, acute kidney injury; AUS, abdominal ultrasound; Cr, creatinine; CT, computed tomography; FDG-PET/CT, fluorine-18 fluorodeoxyglucose positron emission tomography/computed tomography; F, female; M, male; MPO, myeloperoxidase; NA, not available; PAS, periodic acid–Schiff. Sex refers to biological sex as recorded in the medical records.

DWI is an MRI technique that detects the movement of water molecules within tissues. Infiltrative lesions of hematologic malignancies often exhibit high cellular density, which restricts water diffusion and results in high signal intensity on DWI.^8^ In some cases, high signals on DWI may be because of the T2 shine-through effect, in which tissues with intrinsically high T2 signals appear bright on DWI despite the absence of true diffusion restriction. To address this, ADC mapping is used alongside DWI. ADC maps quantify the degree of water diffusion, and areas with true diffusion restriction appear as low signal on the ADC map. When interpreted together, DWI and ADC mapping allow better discrimination of pathologic lesions with high cellularity.^8^

Given the incidence of kidney infiltration and its diagnostic challenges, MRI may offer greater sensitivity. MRI, specifically DWI and ADC mapping, can potentially identify areas of increased cellularity and restricted diffusion. Recently, we reported that DWI is highly sensitive for detecting IgG4-TIN lesions and that DWI findings are useful for predicting the degree of fibrosis in IgG4-TIN.^9^^,^^10^

In the present case, we suspected infiltration of the kidneys in a patient who developed kidney impairment during the course of untreated AML. We hypothesized that, because of high cellular density, infiltrative lesions in the kidney would have high signal intensity on DWI and low signal intensity on ADC mapping, thereby allowing highly sensitive detection. Although no notable findings were observed on CT, multiple mass lesions with high signal intensity on DWI and low signal intensity on the ADC map were scattered throughout the kidneys, which is consistent with our hypothesis. Biopsy confirmed blast infiltration.

There are 3 main advantages of using MRI to detect kidney infiltration in patients with AML. First, it enables the identification of kidney infiltration at an early stage via a noninvasive, relatively low-cost imaging technique that is readily accessible in many facilities. Second, MRI can be performed safely without contrast agents, eliminating the risk of further deterioration of kidney function. Third, identifying lesion sites on MRI before performing a kidney biopsy near the detected lesions reduces the risk of sampling error.

Compared with previous studies summarized in Table 2, the novelty of our case is the use of DWI and ADC mapping to identify infiltration in the kidneys, followed by a kidney biopsy that confirmed the diagnosis and resulted in prompt treatment. This approach led to the successful management of both the patient's AML and his kidney dysfunction. To our knowledge, this is the first case in which kidney infiltration was identified in a patient with AML on the basis of imaging findings.

This study has several limitations. First, it included only 1 patient, indicating a need for future studies with additional patients. Additionally, verifying the accuracy of puncture biopsies of MRI-detected lesions remains challenging. However, based on the principles of MRI, sites with high signal intensity on DWI and low signal intensity on the ADC map are likely to have a relatively high blast density. Performing a biopsy in these areas, as done in this case, helps reduce sampling error using a noninvasive approach. Additionally, the kidney biopsy did not show any significant glomerular abnormalities, and the cause of proteinuria remained unclear.

In conclusion, we present a case of AML with kidney dysfunction in which DWI and ADC mapping detected blast infiltration in the kidneys. This approach facilitated a timely, targeted kidney biopsy and early intervention, with improvement in both AML and kidney function. Larger studies are needed to validate MRI’s diagnostic value and assess its role in standard practice for suspected kidney infiltration in patients with AML.

## References

[1] Luciano R.L., Brewster U.C. (2014). Kidney involvement in leukemia and lymphoma. Adv Chronic Kidney Dis.

[2] Ooi K.Y., Gujadhur A., Pham A., Lukito P., Flanc R., Menahem S. (Aug 2013). Infiltrative acute myeloid leukaemia as a cause of acute kidney injury. Clin Kidney J.

[3] Aratani S., Aburakawa S., Ryotokuji T. (2020). Primary tumor infiltration and severe acute kidney injury in patients with acute myeloblastic leukemia. J Nippon Med Sch.

[4] Chauhan P., Gupta A., Ora M., Agrawal S., Nityanand S. (2021). AML with renal infiltration manifesting as acute renal failure, diagnosed with FDG-PET CT scan: case report. Ann Hematol Oncol.

[5] Aggarwal J., Pathak N.M., Rathore V., Paras Badge R., Sharma A. (2024). Acute kidney injury secondary to leukemic infiltration of the kidneys in m3 acute myeloid leukemia. Indian J Nephrol.

[6] Lee S.W., Kim M.S., Kim Y.J. (2024). Severe acute kidney injury associated with transformation of chronic myelomonocytic leukemia into acute myeloid leukemia: a case report. J Clin Med.

[7] Lahoti A., Kantarjian H., Salahudeen A.K. (2010). Predictors and outcome of acute kidney injury in patients with acute myelogenous leukemia or high-risk myelodysplastic syndrome. Cancer.

[8] Goyal A., Sharma R., Bhalla A.S., Gamanagatti S., Seth A. (2012). Diffusion-weighted MRI in assessment of renal dysfunction. Indian J Radiol Imaging.

[9] Suenaga A., Sawa N., Ikuma D. (2023). Immunoglobulin G4-related tubulointerstitial nephritis with simultaneous resolution of plasma cell infiltration and fibrosis after steroid treatment. Intern Med.

[10] Suenaga A., Oba Y., Ikuma D. (2025). Relationship between MRI findings and renal histopathology in IgG4-related tubulointerstitial nephritis. Mod Rheumatol.

[11] Pickhardt P.J., Lonergan G.J., Davis C.J., Kashitani N., Wagner B.J. (2000). From the archives of the AFIP. Infiltrative renal lesions: radiologic-pathologic correlation. Armed Forces Institute of Pathology. Radiographics.

